# Can Cities Activate Sleeper Species and Predict Future Forest Pests? A Case Study of Scale Insects

**DOI:** 10.3390/insects11030142

**Published:** 2020-02-25

**Authors:** Steven D. Frank, Michael G. Just

**Affiliations:** Department of Entomology and Plant Pathology, Campus Box 7613, North Carolina State University, Raleigh, NC 27695, USA; mjust@ncsu.edu

**Keywords:** global change, latent invasive species, urban, warming

## Abstract

Sleeper species are innocuous native or naturalized species that exhibit invasive characteristics and become pests in response to environmental change. Climate warming is expected to increase arthropod damage in forests, in part, by transforming innocuous herbivores into severe pests: awakening sleeper species. Urban areas are warmer than natural areas due to the urban heat island effect and so the trees and pests in cities already experience temperatures predicted to occur in 50–100 years. We posit that arthropod species that become pests of urban trees are those that benefit from warming and thus should be monitored as potential sleeper species in forests. We illustrate this with two case studies of scale insects that are important pests of urban trees in parts of the US. *Melanaspis tenebricosa* and *Parthenolecanium quercifex* are geographically native to the US but take on invasive characteristics such as higher survival and reproduction and become disconnected from natural enemies on urban trees due to the urban heat island effect. This allows them to reach high densities and damage their host trees. *Parthenolecanium quercifex* density increases up to 12 times on urban willow oaks with just 2 °C of warming due to higher survival and adaptation to warmer temperatures. The urban heat island effect also creates a phenological mismatch between *P. quercifex* and its parasitoid complex, and so egg production is higher. *Melanaspis tenebricosa* density can increase 300 times on urban red maples with 2.5 °C of warming. This too is due to direct effects of warmer temperatures on survival and fecundity but *M. tenebricosa* also benefits from the drought stress incurred by warmer urban trees. These effects combine to increase *M. tenebricosa* density in forests as well as on urban trees at latitudes higher than its native range. We illustrate how cities provide a unique opportunity to study the complex effects of warming on insect herbivores. Studying pestilent urban species could be a pragmatic approach for identifying and preparing for sleeper species.

Sleeper species are innocuous native or naturalized species that exhibit invasive characteristics and become pests in response to environmental change [1]. Climate warming is expected to increase arthropod damage in forests, in part, by transforming innocuous herbivores into severe pests: awakening sleeper species [2,3,4]. Several native insect herbivores have already become invasive forest pests. For example, warming has transformed the mountain pine beetle (*Dendroctonus ponderosae* (Hopkins)) from an intermittent outbreak pest to an invasive pest by increasing winter survival, voltinism, and geographic range (latitude and altitude) [5,6,7,8]. In these newly invaded areas, mountain pine beetles encountered naive host trees, often stressed by climate, amplifying their lethality [9,10]. Southern pine beetle (*Dendroctonus frontalis* Zimmermann), native to the southern US, and pine processionary moth (*Thaumetopoea pityocampa* Denis and Schiffermüller), native to the Mediterranean region, have also spread and become damaging pests as warmer winters have permitted their survival at higher latitudes [11,12]. Trees host a remarkable number of herbivorous arthropod species. For example, *Quercus* and *Acer* trees in the mid-Atlantic USA host 535 and 297 Lepidoptera species, respectively [13]. In the British Isles, *Quercus* trees host over 400 insect species [14]. With so many native or naturalized insect species on a given tree and continued warming, how do we identify and prepare for sleeper species?

Cities are warmer than adjacent natural areas due to the urban heat island (UHI) effect [15]. Urban trees live at these warmer temperatures and often have more arthropod pests than trees in natural areas [16,17,18,19]. The UHI effect can increase herbivore populations directly by increasing their development rate, voltinism, winter survival, or fecundity [18,20,21,22,23]. The UHI effect can increase herbivore populations indirectly by increasing host plant quality or reducing regulation by natural enemies [18,20]. Warmer temperatures are not the only reason background herbivores become invasive pests on urban plants but the UHI effect is a major driver structuring arthropod communities [24,25,26,27,28]. Cities provide opportunities to study the complex effects of warming now, even when considering for environmental differences, such as landcover, disturbance, soil moisture, and natural enemies, between cities and natural habitats (as reviewed in [29]). Thus, cities may help predict the response of plants and animals to climate change [29]. Pestiferous species that thrive on urban trees may be sleeper species that will become invasive forest pests with continued warming.

Invasive species often have inherent or plastic traits that increase their fitness, competitiveness, or reproductive rate as compared to non-invasive species [30,31,32]. There is no set definition of what characteristics make a species invasive, but rather a collection of characteristics that are common to species considered invasive. These characteristics are usually applied to non-native species and include sexual and asexual reproduction, high reproductive and dispersal rates, phenotypic plasticity or adaptation, release from enemies, rapid geographic spread, and negative effects on human interests and biodiversity [33,34,35,36,37,38]. Warming can promote or activate these traits in some herbivorous arthropods [34,39,40]. For example, the diamondback moth (*Plutella xylostella* (L.)) is a long-distance disperser and is a cosmopolitan agriculture pest [41]. Climate warming has increased its dispersal ability as warmer air currents facilitate longer distance dispersal, allowing it to be pestilent in areas where it could not successfully overwinter [42,43], though establishment is occurring in some of these areas as they become warmer [44]. With warming, the eastern larch beetle (*Dendroctonus simplex* Le Conte) has become more pestilent in its native range [45], with some populations changing from univoltine to bivoltine during warmer years [46]. Species that display invasive phenotypes (i.e., those phenotypes that include characteristics considered invasive) due to urban warming may be the sleeper species that will become invasive forests pests with continued climate warming e.g., [47,48].

The effects of urban warming have been particularly well studied for two scale insect taxa that are urban tree pests, gloomy scale, *Melanaspis tenebricosa* (Comstock) and oak lecanium scale *Parthenolecanium quercifex* (Fitch). These scale species develop invasive traits, proliferate, and become chronic urban tree pests due to the UHI effect [49,50,51]. Other scale species on the same trees under the same conditions do not display invasive phenotypes or become pests [52,53]. *Melanaspis tenebricosa* and *P. quercifex* are consistent with characterizations of sleeper species as they are innocuous native species that become pests with environmental change, here, the UHI effect. Moreover, we suspect that activation of these species and other species by the UHI effect forewarns their emergence as forest pests as the climate warms. Here, we present case studies of these scale insect species to describe how they have become invasive in cities and how these characteristics could predict their eventual emergence as forest pests. We focus on a few traits associated with invasive species that could be and have been affected by warming: (1) phenotypic plasticity that increases fitness and abundance, (2) the uncoupling of interactions with local natural enemies, and (3) geographic range expansion (Table 1). We review research on these species and demonstrate the primacy of warming in altering these traits—leading to high densities and virulence of these pests—as compared to other environmental factors. We also discuss evidence for their potential to spread into natural forests with climate warming and how this approach could be used more generally.

## 1. Oak Lecanium Scale and Willow Oaks

**Figure 1:**
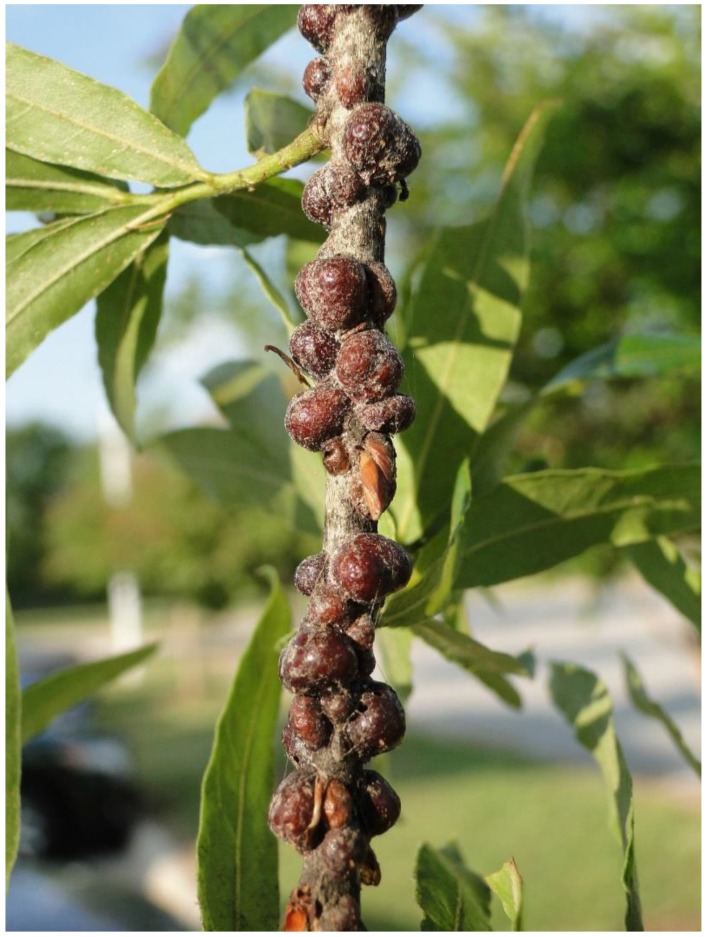
Willow oak (*Quercus phellos*) branch heavily infested with oak lecanium scale (*Parthenolecanium quercifex*) ovisacs. Photo: Clyde Sorenson, North Carolina State University.

*Natural history*. *Parthenolecanium quercifex* is native to the eastern US and feeds primarily on oak trees (Figure 1). They feed on phloem from leaves or branches depending on their life stage. *Parthenolecanium quercifex* are univoltine and adult females begin oviposition in late spring [62]. During this period, the female swells and arches her test to produce a hemispherical container for the eggs (i.e., ovisac). *Parthenolecanium quercifex* produce up to 3000 eggs over a period of approximately 4 weeks. Eggs typically hatch within a week. Crawlers (i.e., first instars) migrate from ovisacs to leaves where they feed through summer. In fall, they molt to second instars and migrate back to tree stems. *Parthenolecanium quercifex* overwinter as second instars and develop into adults in early spring.

*Parthenolecanium quercifex* feeds on many oak species [63] but is a primary pest of willow oak trees in the eastern US [49]. The native distribution of willow oaks in eastern US forests is from New Jersey to northern Florida and they are among the most planted trees in US cities throughout the southeastern and mid-Atlantic states [64]. Willow oaks host diverse arthropod communities, including many specialist and generalist herbivores [63]. Among these are at least 12 scale species and many other related hemipterans, including the congener, *Parthenolecanium corni*, which is not native to the US but often co-occurs with *P. quercifex* on urban trees. The species are indistinguishable during all but the first instar [65]. Therefore, research has often reported their combined abundance. Severe infestations of *P. quercifex* can reduce tree growth especially when combined with other stressors like drought [59]. *Parthenolecanium quercifex* also produce honeydew that coats leaves and other surfaces and is a substrate for sooty molds.

*Relationship of warming and density*. *Parthenolecanium quercifex* density is generally greater on urban willow oaks than willow oaks in forests. The relationship between *P. quercifex* density and temperature has been explored with observational and manipulative experiments. In one experiment, a georeferenced urban tree database and Landsat surface temperature data were used to select willow oak street trees from the warmest and coolest locations in Raleigh, NC, US (35°47′15.8′’ N, 78°38′39.3′’ W) [54]. Temperature was also recorded throughout the experiment with in situ data loggers. Scale density was recorded throughout the year to assess density at different life stages: ovisacs in spring, first instars on leaves in summer, and second instars on twigs in fall. The density of each life stage was 8–12 times higher on the warm trees than cool trees [54]. No differences were found in natural enemy abundance or percent parasitism of adults, suggesting an important role of temperature on scale density. Similar observational experiments using street trees produced comparable relationships between temperature and *P. quercifex* density and natural enemies [51,59]. Willow oaks, and other oak species, host other scale species [49,63], but only *P. quercifex* density increased with temperature in this study.

The relationship between *P. quercifex* density and temperature was further confirmed with experiments comparing street trees to forest trees at the same and different latitudes [57]. Scale density, temperature, and natural enemy community were recorded for street trees in Raleigh and 3.8 degrees of latitude higher in Newark, DE, US (39°41′1.4′’ N, 75°44′58.8′’ W). Mean canopy temperature of street trees in Raleigh was 0.8 °C warmer than that of forest trees and street trees had over eight times more *P. quercifex*. Newark had a mean temperature during the observational period 3.1 °C below that of Raleigh, similar to the difference between Raleigh street and forest trees. Overall, Newark trees had seven times less scales than Raleigh street trees, similar to the differences between Raleigh street and forest trees. There were no scales detected on forest trees in Newark. This suggests that the cooler background temperature of Newark, due to latitude, limited *P. quercifex* from reaching pest densities on urban trees and that there could be a threshold temperature at which *P. quercifex* switch from background to pestiferous herbivores [57]. The mechanisms driving this change in density need to be uncovered because they are important for understanding the activation of sleeper species and potential for scales to reach high densities in locations that are currently at low densities.

*Invasive trait: phenotypic change*. High survival rate is a mechanism that increases the population growth, spread, and persistence of invasive species [66]. Growth chamber experiments were conducted to determine whether greater survival is a mechanism for *P. quercifex* density on trees under warm conditions [59]. Greenhouse and growth chamber experiments help control for potentially confounding biotic and abiotic factors that may differ between urban and natural areas, such as soil type, plant cover, natural enemy presence and activity, water availability, and pollutants. *Parthenolecanium quercifex* ovisacs collected from urban trees were attached to willow oak saplings in large growth chambers (9 m^3^). Saplings were grown in either warm or cool chambers under high or low water treatments. The cool chamber temperature was set to the monthly mean outdoor temperature (30 year normals) [67]. The warm chamber was maintained at 4 °C higher than average to emulate the UHI effect. First instar scales had approximately 20% greater survival in the month after eclosion in the warm chambers than cool chambers. Water stress did not have a significant effect on *P. quercifex* survival [59]. Crawlers are the most vulnerable stage of scale insects and their survival is often low. Thus, greater survival during this important life stage could affect population growth in field populations.

Scale insects can adapt to new hosts or abiotic conditions through genetic or plastic phenotypic changes that increase their fitness [68,69,70,71]. A greenhouse experiment was conducted to determine whether *P. quercifex* collected from warm trees were adapted to, and gained an advantage in, warmer conditions absent other urban environmental characteristics [54]. In this experiment, willow oak saplings were grown in a warm greenhouse maintained at 36/32 °C (day/night) and in a cool greenhouse that was 32/28 °C. *Parthenolecanium quercifex* ovisacs were collected from trees in warm and cool locations in Raleigh and attached to chamber saplings in a factorial design that included scale thermal origin (warm or cool) with chamber temperature (warm or cool). After three months, the density of scales from ovisacs from cool trees was similar in warm and cool chambers. The density of scales from ovisacs from warm trees was over three times greater in warm chambers than in cool chambers. This suggests scales from warm trees had some plastic or genetic adaptation to warmer conditions that increased their survival and densities. Warming scales from cool trees did not produce the invasive phenotype (i.e., survival was not greater) in the single season of the experiment [54]. Multigeneration experiments are needed to determine whether scales acquire this phenotype through maternal effects or other mechanisms.

The hypothesis that scales in cool climates develop invasive phenotypes with climate warming can be tested with reciprocal transplant and common garden experiments that also help alleviate the confounding effects of non-temperature urban conditions on scale responses. A common garden experiment was conducted to help determine whether *P. quercifex* at high latitudes could develop invasive phenotypes that included higher survival and density as the climate warmed [57]. *Parthenolecanium quercifex* ovisacs were collected from Newark, DE and from Raleigh, NC in spring and then attached to young willow oaks planted in a common garden in Raleigh. Raleigh has a mean temperature that is approximately 2.8 °C warmer than Newark. Thus, this design simulated global estimates of climate warming over the next 50–100 years [72]. Scales from Newark reached densities more than three times greater than scales from Raleigh and these densities are not often recorded from urban trees in Newark [57]. This suggests that climate warming could activate *P. quercifex*—through mechanisms such as increased size, fecundity, or survival—in areas where it currently exists as a background herbivore.

*Invasive trait: uncoupled natural enemy interactions*. Many native and exotic species become invasive because they are uncoupled from natural enemies [73]. Climate warming can also disrupt trophic interactions by changing natural enemy behavior, abundance, or community composition [74,75,76,77]. Warming can also create phenological mismatches between herbivores and natural enemies which are predicted to occur frequently with climate warming [78,79,80].

Field research on *P. quercifex* has shown that its natural enemy communities are similar between trees in warm and cool areas as well as between forest and street trees [54,57]. In fact, natural enemies, like parasitoids, are often more abundant in trees with more scales [19,21,81]. Percent parasitism of *P. quercifex*, assessed by dissections, was also similar between scales on warm and cool street trees in Raleigh [54]. Taken together this evidence suggests that warming increased scale density directly, as discussed above, rather than indirectly by altering interactions with natural enemies. However, a field experiment uncovered a phenological mismatch between *P. quercifex* and parasitoids at a key stage in its life cycle that contributed to greater reproductive success and population growth [51]. *Parthenolecanium quercifex* produce eggs in spring and around the time of oviposition, several parasitoids become active and parasitize the adult female scales and lay eggs that develop within the scale body or ovisac [82]. Parasitism can reduce or prevent oviposition depending when it occurs during development. In this field experiment, female scales from street trees in warm sites and sites approximately 2.5 °C cooler were collected weekly, starting prior to oviposition. At each collection, the proportion of egg-producing scales and the proportion of parasitized scales was recorded. Scales on warm trees produced eggs approximately two weeks earlier than scales on cool trees. Scales were parasitized at similar rates on warm and cool trees but scales on warm trees produced more eggs before they were parasitized. Thus, egg production by unparasitized scales was similar on warm and cool trees but egg production was doubled for parasitized scales in warm trees [51]. Greater egg production due to this mismatch coupled with higher survival of first instars on warm trees could increase population growth, an invasive trait, and, thus, make *P. quercifex* an invasive species.

## 2. Gloomy Scale and Red Maples

**Figure 2:**
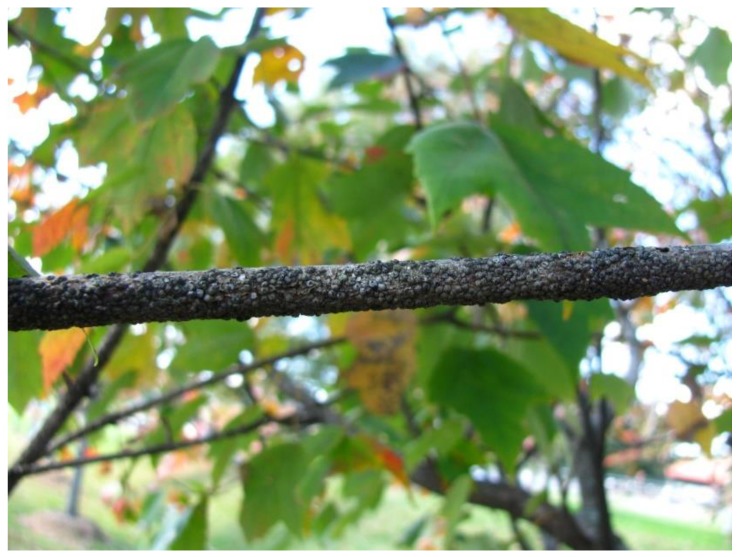
Red maple (*Acer rubrum*) branch heavily infest with gloomy scale (*Melanaspis tenebricosa*). Photo: Elsa Youngsteadt, North Carolina State University. The small bumps covering the bark are scale tests. See Dale, A.G., Frank, S.D., 2014. Urban Warming Trumps Herbivore Enemies. Bull. Ecol. Soc. Am. 95, 252–256 for additional images.

*Natural history*. Gloomy scale, *Melanaspis tenebricosa*, is native to the southeastern US (Figure 2). It feeds primarily on red maple and other maples though it can be found on other species, including tulip poplar (*Liriodendron tulipifera*), hackberry (*Celtis occidentalis*), sweet gum (*Liquidambar styraciflua*), and holly (*Ilex americana*) [16,83]. *Melanaspis tenebricosa* is univoltine and lives on tree bark where it feeds on fluid from xylem or parenchyma cells [84]. Mated females overwinter and begin oviposition in late spring. Each scale produces up to 5–7 eggs per day over 6–8 weeks [21,85]. Crawlers (i.e., first instars) leave the test and settle on branches or the trunk, often within a few centimeters of the mother. At these settling sites, a crawler molts, builds its test, molts again, and expands its test. After the last molt in late summer, winged males emerge to mate with females—after which, the males die [83].

In the southeastern US, *M. tenebricosa* are primarily a pest of planted red maples along streets and in urban landscapes. They were identified as the most important pest of urban red maples in 1912 [16] and remain so today [49]. Yet, red maples are hosts to specialist herbivores such as painted maple aphids (*Drepanaphis acerifoliae* (Thomas)) and maple spider mites (*Oligonychus aceris* (Shimer)), generalist herbivores, and at least 16 other scale species [63,84,86]. In the southeastern US, these other herbivores rarely become chronic pests of red maples in urban or rural forests. *Melanaspis tenebricosa* densities increase rapidly on street trees 6 to 10 years after planting [50]. They can reach densities exceeding 70 scales per cm [87] and encrust branches and trunks, giving trees a dark gray ‘gloomy’ appearance. Heavy *M. tenebricosa* infestations cause dieback of small branches and sparse canopies. Eventually, larger branches may die with trees acquiring generally poor condition and appearance [55].

*Relationship of warming and density*. *Melanaspis tenebricosa* density is generally greater on urban trees than trees in natural areas [16,56,87]. Gloomy scale density is positively correlated with air temperature and the amount of impervious surface cover around trees [55]. Initial research to understand *M. tenebricosa* infestation of urban trees assessed scale and natural enemy density and community composition on trees that varied in canopy temperature and the amount of circumjacent impervious surface over two years. The trees spanned a temperature gradient of approximately 2.5 °C as measured within their canopies. Natural enemy density and community composition did not differ among the trees and were not a significant predictor of scale density in path analyses [21]. The strongest path indicated that impervious surface cover increased temperature which, in turn, increased scale density. Subsequent research on urban red maples corroborated these results [53,56,61,87].

The benefits of warming for *M. tenebricosa* were also assessed by comparing urban and forest trees in Raleigh and 3.8 degrees of latitude higher in Newark, DE [56]. *Melanaspis tenebricosa* density was measured on solitary trees planted in landscapes and on trees growing naturally on the edge and interior of adjacent forests. In Raleigh, landscape trees were 1.5 °C warmer than forest edge or interior trees and had three orders of magnitude more scales. In Newark, which has a mean temperature that is 2.8 °C cooler than Raleigh, forest and landscape trees had similar, low scale densities as compared to Raleigh street trees. To separate the effects of temperature from differences in edaphic conditions (e.g., water availability, compaction, nutrients), an experiment was conducted with potted red maple saplings. Saplings were planted in bark and sand potting mix and infested with gloomy scales. The following year the potted trees were placed next to a subset of the landscape and forest trees in late spring (May 2014) before the *M. tenebricosa* oviposition period and moved to nursery pad after oviposition ended (July 2014). Potted trees were watered three times each week. At the end of the second summer (September 2016), scale density was five times greater on potted trees next to landscape trees than on forest trees, indicating a benefit of the higher temperature in landscapes. Additionally, parasitoid and predator abundances were four times greater in landscapes suggesting natural enemies were not responsible for lower *M. tenebricosa* density in forests [56].

*Invasive trait: phenotypic change*. Common phenotypic changes observed in invasive species are greater size or fecundity [88,89,90]. These changes can be due to favorable abiotic conditions, higher quality food, less competition, or other factors [66,91,92,93]. In an observational experiment, *M. tenebricosa* were collected from urban trees that spanned a temperature gradient of approximately 2.5 °C [21]. The number of embryos within scales was positively correlated with tree canopy temperature in each of four collections that were each approximately a week apart. Scale size (i.e., body length) was also positively correlated to temperature in this experiment and scales were up to 30% larger in trees from warmer locations.

Tree quality for herbivores can increase or decrease with water stress and the effects of water stress on scales has been equivocal. Research on white peach scale (*Pseudaulacaspis pentagona* (Targioni)) found water stress reduced population growth and survival [94]. However, elongate hemlock scales (*Fiorinia externa* Ferris) became more abundant on trees subjected to water stress [95]. Some research on *M. tenebricosa* found that although scale density was positively correlated with canopy temperature, impervious surface cover was a stronger predictor of scale density [96]. Impervious surface cover affects temperature but also the soil moisture available to trees [97]. As such, tree water stress, measured as xylem water potential, was found to be correlated with impervious surface cover around red maples in a different study [55].

A field experiment was conducted to disentangle the effects of temperature and water stress on *M. tenebricosa* density and fecundity [98]. Pairs of red maple street trees were selected at 30 sites in Raleigh that spanned a range of impervious surface cover and air temperature. One tree in each pair was assigned to a ‘watered’ treatment and received supplemental water through the summer for two years, ‘unwatered’ trees received no supplemental water. Thus, the temperature which increases with impervious surface could be separated from water stress which also increases with impervious surface. Slow release irrigation bags were placed on each tree in the watered treatment and filled twice each week from June through August, when temperature and water stress are greatest in Raleigh, NC. *Melanaspis tenebricosa* fecundity was measured after a two-year experimental duration. There were additive main effects of temperature and watering in which the warmest unwatered trees had approximately 17% more embryos than warm watered trees but these had 65% more than cool watered trees [98]. Thus, higher temperature and tree water stress, both of which will increase with climate change, work in concert to produce the invasive phenotypes of higher fecundity and population growth.

*Melanaspis tenebricosa* survival and establishment also increases at warmer temperatures. The proportion of scales that survived from one year to the next was greater on trees that were 2.5 °C warmer than cooler trees in Raleigh, NC as measured by the ratio of adults from one generation to the next [21]. In another study, *M. tenebricosa* establishment and rate of scale accumulation was greater on trees surrounded by greater proportions of impervious surface [50]. A growth chamber experiment was used to test the effect of temperature, while limiting or removing the effect of other uniquely urban conditions, on the establishment and survival of *M. tenebricosa*. Scales in the warmer chambers developed faster, reached higher densities, and had greater survival [58]. These results indicate that the benefits of warming on early stage settlement and survival are important in reaching high scale densities on already-warmer urban trees and may also benefit scales when forests warm.

*Invasive trait: range expansion*. Invasive species are often characterized by geographic range expansion into previously unoccupied areas [99]. In 1922, the range of *M. tenebricosa* was delimited to include states from Maryland in the north to Florida in south along the east coast of the USA and westward from Missouri to Texas. Recent surveys e.g., [53,85] have found them throughout the southeastern US, in agreement with the Metcalf (1922) map. We surveyed red maple street trees in Boston, MA, Queens, NY, Philadelphia, PA, Newark, DE, and Baltimore, MD, along the east coast of the US. Each of these cities is north of the 1922 *M. tenebricosa* distribution. These surveys revealed *M. tenebricosa* in Queens (1 of 40 trees), Philadelphia (4 of 7 trees), Newark (18 of 35 trees), and in Baltimore (6 of 33 trees) [57,60]. No *M. tenebricosa* were found in Boston (39 trees). In addition, we surveyed red maples in Asheville, NC (elevation: 650 m) and found *M. tenebricosa* on 12 of 35 trees [61] in a county not previously recorded as having *M. tenebricosa* [83]. These findings suggest *M. tenebricosa* can exist at latitudes north or elevations greater than its previous distribution likely due to combined effects of the UHI effect and climate warming that reduce winter temperature extremes and reduces the frequency of lethal cold events [100]. These observations could be followed up with research to identify the mechanisms (e.g., thermal tolerance [101]) related to this range movement in cities and whether *M. tenebricosa* has spread to rural or natural forests in the last century.

## 3. Cities Simulate Climate Change

There is evidence that cities can have similar effects on plant and animal species as those predicted by outdoor warming experiments and experiments in growth chambers [29]. The waxy tests of *M. tenebricosa* remain on plants after the insects die and, thus, are preserved on herbarium specimens. Youngsteadt et al. (2014) inspected 342 red maple herbarium specimens collected in forests from 1895 to 2011 from North Carolina, South Carolina, and Georgia. *Melanaspis tenebricosa* were counted on each specimen and temperature was estimated for the collection location by year of collection. They also collected twigs and temperatures from urban red maples. The modeled response of herbarium and urban scale to temperature was similar in shape but different in magnitude with urban scales found at six times greater density. *Melanaspis tenebricosa* was found on approximately 29% of sites from herbarium specimens. The frequency of *M. tenebricosa* on samples was greatest in historically warm periods of the 20th century and lower in historically cool periods. This documents fluctuation in scale occurrence with climate in forests and the potential for extended warming to make *M. tenebricosa* more common in forests. Warming in the past 40 years has been greater in magnitude and duration than the other warm periods analyzed initially. To determine how recent extended warming affected *M. tenebricosa* density, 20 forest sites where herbarium specimens had been collected were revisited. Random twigs were collected from red maples at each site and processed for storage that matched the procedures of the herbarium samples. At 16 of the sites, scale density had increased compared to two that were the same and two that declined [87]. Within the *M. tenebricosa* native range, warming increases scale occurrence and density in forests where they generally occur at low densities, thus providing evidence for potential future spread into forests. Moreover, recent reviews have evaluated the potential for and evidence of range expansion for plant pests and pathogens with warming [102,103].

## 4. Conclusions

Most herbivorous arthropods are not pests. They are background herbivores that contribute to the biodiversity and functioning of ecosystems. Yet, native and exotic sleeper species are a growing threat to forests and other ecosystems with continued warming. Here, we illustrated how cities provide a unique opportunity to study the complex effects of warming on insect herbivores. The two scale insect species presented show multiple invasive traits on urban trees due to the UHI effect. The effects of warming were also detected in laboratory experiments and forest observations supporting the assertion that warming is driving the scale density and the phenotypic changes observed rather than other urban factors. Many other scale species are chronic or occasional pests on urban trees and temperature is unlikely to be the primary driver of every species. Geographically native species such as tulip tree scale, obscure scale, pine needle scale, and others in the US are important pests of urban trees though mechanisms that drive these pests are not well established [19,49,104]. Tree stress, depauperate natural enemy communities, insecticides, and fertilizer have all been attributed to high densities of scale insects and other pests on urban trees [18,19,94,105,106]. Connections between temperature and scale outbreaks have been described relatively recently but may help unify some other proposed mechanisms since, for example, heat is caused by impervious surfaces that also fragment and reduce natural enemy habitats and cause tree stress [20].

Scales and their relatives can kill or sicken trees across vast geographic areas [107,108], changing the structure and functions of forest ecosystems [109,110,111]. Warming can benefit many of these pests directly and indirectly increasing survival, reproduction, and population growth [112]. Higher fitness for pests combined with potentially greater stress of their host trees is expected to increase the spread and pestilence of geographically native and exotic species. For example, hemlock woolly adelgids, elongate hemlock scale, and the scales associated with beech bark disease benefit from warmer winters at high latitudes where they would otherwise not survive [113,114,115,116]. The geographic ranges of many other scale and non-scale tree pests are restricted by cold temperatures. These species would be expected to survive or even thrive in cities at latitudes higher than where they occur in forests. This is what we see in gloomy scale but also pine processionary moth which survives in the Paris, FR heat island although it is north of the natural expansion front [117]. The UHI effect benefits pine processionary moth caterpillars by increasing development rate and winter survival [118].

Not all arthropods benefit from higher temperatures. Negative effects of the UHI effect have been documented for many non-pest arthropod species, including some ants, bees, butterflies, and spiders [24,27,28,119]. However, negative effects of warming have also been documented for some pest species, including *Adelges tsugae* (Annand) (hemlock wooly adelgid) [120], *Lymantria dispar* L. (gypsy moth) [121], *Matsucoccus matsumurae* (Kuwana) (pine best scale) [122], and *M. tenebricosa* [101] where studies on each have shown evidence for range stasis or contraction with warming. Thus, the UHI effect can not only portend the awakening of pest species that benefit from warming but also identify species destine to decline with climate warming. Identifying and studying pestilent urban species could be a pragmatic approach for identifying and preparing for sleeper species [29].

Further research is needed to determine whether this approach applies broadly to arthropods, other ectotherms, plants, and even pathogens. Research in some of these systems has identified species that are considered latent invasive or pest species (e.g., insects [123], pathogens [124]), where they remain quiescent until conditions are more suitable. A pragmatic starting point for research to identify other potential sleeper species would be to examine those insects that are already pests or more abundant in cities. For example, *Thyridopteryx ephemeraeformis* (Haworth) (bagworms) are an urban tree pest whose distribution is limited by cold temperatures [125,126], but have demonstrated greater survival in areas with greater impervious surface [127]. In Europe, *Eotetranychus tiliarum* Hermann (lime mite) had four times greater abundance and three times greater fecundity on *Tilia* sp. (linden) trees that were on the sunnier side of the street as compared to mites on trees on the shadier side [128]. The overwintering survival of *Hornadaula anisocentra* Meyrick (mimosa webworm) was greater on trees or branches that were nearer buildings and more protected from the cold [23]. Given the benefits of the UHI effect on these species, their potential to be future forest pests may be similar to the scales we discuss here. However, importantly, future monitoring will document whether gloomy scale, oak lecanium scale, and other potential sleeper species actually become forest pests as predicted.

## Figures and Tables

**Table 1:** Summary of the effects of urban warming on gloomy scale (*Melanaspis tenebricosa*) and oak lecanium scale (*Parthenolecanium quercifex*) invasiveness.

Invasive Trait	*M. tenebricosa*	*P. quercifex*
Increased reproductive rate	Greater embryo production on warmer trees [21]	Greater ovisac density on warmer trees [54]
Increased density	Scale density 200 times greater on urban trees with 2.5 °C of warming [55];	Scale density 8–12 times greater on urban trees with 2.5 °C of warming [54];
	five times more abundant on street trees than forest trees [56]; greater accumulation [50]	seven times more abundant at low than high latitudes and on street than forest trees (Δ 3.8 °C latitude) [57]
Plastic or genetic adaptation/phenotypic change	Scales are 30% larger on warmer urban trees [21]; greater survival [21,58]; greater establishment [50,58]	Crawler survival 20% greater at higher temperatures [58,59]; three times greater density [58,59]; three times greater density in warmed common garden [57]
Enemy release	No increase in total natural enemy density [21] or parasitoid density [56] with urban warming or natural enemy density tracks scale density [56]	Phenological mismatch between scales and parasitoids on warm urban trees [51]
Range expansion	Northward expansion [57,60]; found at greater elevations [61]	no observations available
